# Tetra­kis(4-methyl-2-thien­yl)tin(IV)

**DOI:** 10.1107/S1600536808032790

**Published:** 2008-10-15

**Authors:** Quai Ling Yap, Kong Mun Lo, Seik Weng Ng

**Affiliations:** a Department of Chemistry, University of Malaya, 50603 Kuala Lumpur, Malaysia

## Abstract

The mol­ecule of the title compound, [Sn(C_5_H_5_S)_4_], lies on a special position of 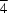 site symmetry. The Sn^IV^ atom shows a slightly distorted tetra­hedral coordination.

## Related literature

For the structure of tetra­kis(2-thien­yl)tin, see: Karipides *et al.* (1977 ▶). For the synthesis, see: Kumar Das *et al.* (1987 ▶).
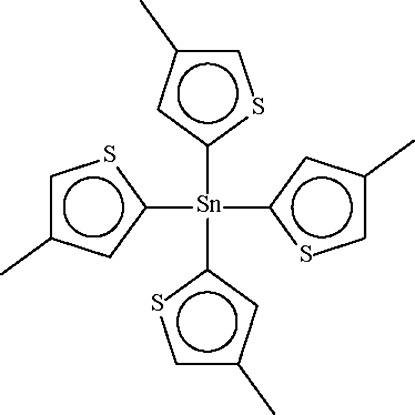


## Experimental

### 

#### Crystal data

[Sn(C_5_H_5_S)_4_]
*M* *_r_* = 507.29
Tetragonal, 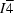

*a* = 11.6286 (9) Å
*c* = 7.5918 (6) Å
*V* = 1026.6 (1) Å^3^
*Z* = 2
Mo *K*α radiation
μ = 1.65 mm^−1^
*T* = 100 (2) K
0.30 × 0.25 × 0.10 mm

#### Data collection

Bruker SMART APEX diffractometer
Absorption correction: multi-scan (*SADABS*; Sheldrick, 1996 ▶) *T*_min_ = 0.637, *T*_max_ = 0.852
2948 measured reflections
1151 independent reflections
1149 reflections with *I* > 2σ(*I*)
*R*_int_ = 0.014

#### Refinement

*R*[*F* ^2^ > 2σ(*F* ^2^)] = 0.012
*wR*(*F* ^2^) = 0.028
*S* = 1.01
1151 reflections
58 parameters
H-atom parameters constrained
Δρ_max_ = 0.45 e Å^−3^
Δρ_min_ = −0.21 e Å^−3^
Absolute structure: Flack (1983 ▶), 513 Friedel pairs
Flack parameter: 0.005 (14)

### 

Data collection: *APEX2* (Bruker, 2007 ▶); cell refinement: *SAINT* (Bruker, 2007 ▶); data reduction: *SAINT*; program(s) used to solve structure: *SHELXS97* (Sheldrick, 2008 ▶); program(s) used to refine structure: *SHELXL97* (Sheldrick, 2008 ▶); molecular graphics: *X-SEED* (Barbour, 2001 ▶); software used to prepare material for publication: *publCIF* (Westrip, 2008 ▶).

## Supplementary Material

Crystal structure: contains datablocks global, I. DOI: 10.1107/S1600536808032790/ci2670sup1.cif

Structure factors: contains datablocks I. DOI: 10.1107/S1600536808032790/ci2670Isup2.hkl

Additional supplementary materials:  crystallographic information; 3D view; checkCIF report

## Figures and Tables

**Fig. 1.**
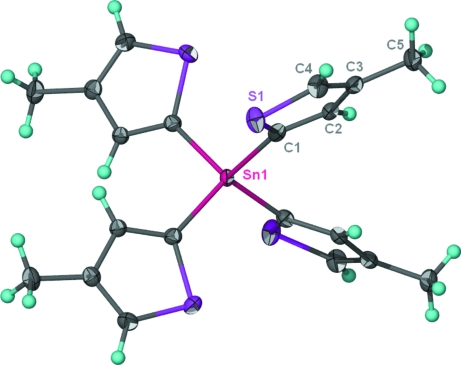
Displacement ellipsoid plot (Barbour, 2001) of [Sn(C5H5S)4] at the 70% probability level. H atoms are drawn as spheres of arbitrary radii.

## supplementary crystallographic information

### Experimental

The title compound was synthesized as reported previously (Kumar Das *et al.*, 1987). Single crystals were obtained upon recrystallization from chloroform.

### Refinement

H-atoms were placed in calculated positions (C-H = 0.95-0.98 Å) and were included in the refinement in the riding model approximation, with *U*(H) set to 1.2–1.5*U*_eq_(C).

### Crystal data

**Table:**

[Sn(C_5_H_5_S)_4_]	*D*_x_ = 1.641 Mg m^−^^3^
*M**_r_* = 507.29	Mo *K*α radiation, λ = 0.71073 Å
Tetragonal, *I*4	Cell parameters from 2958 reflections
Hall symbol: I -4	θ = 2.5–28.3°
*a* = 11.6286 (9) Å	µ = 1.65 mm^−^^1^
*c* = 7.5918 (6) Å	*T* = 100 K
*V* = 1026.6 (1) Å^3^	Prism, colourless
*Z* = 2	0.30 × 0.25 × 0.10 mm
*F*(000) = 508	

### Data collection

**Table:**

Bruker SMART APEX diffractometer	1151 independent reflections
Radiation source: fine-focus sealed tube	1149 reflections with *I* > 2σ(*I*)
graphite	*R*_int_ = 0.014
ω scans	θ_max_ = 27.5°, θ_min_ = 2.5°
Absorption correction: multi-scan (SADABS; Sheldrick, 1996)	*h* = −15→14
*T*_min_ = 0.637, *T*_max_ = 0.852	*k* = −15→13
2948 measured reflections	*l* = −9→8

### Refinement

**Table:**

Refinement on *F*^2^	Secondary atom site location: difference Fourier map
Least-squares matrix: full	Hydrogen site location: inferred from neighbouring sites
*R*[*F*^2^ > 2σ(*F*^2^)] = 0.012	H-atom parameters constrained
*wR*(*F*^2^) = 0.028	*w* = 1/[σ^2^(*F*_o_^2^) + (0.0145*P*)^2^] where *P* = (*F*_o_^2^ + 2*F*_c_^2^)/3
*S* = 1.01	(Δ/σ)_max_ = 0.001
1151 reflections	Δρ_max_ = 0.45 e Å^−^^3^
58 parameters	Δρ_min_ = −0.21 e Å^−^^3^
0 restraints	Absolute structure: Flack (1983), 513 Friedel pairs
Primary atom site location: structure-invariant direct methods	Flack parameter: 0.005 (14)

### Fractional atomic coordinates and isotropic or equivalent isotropic displacement parameters (Å^2^)

**Table:**

	*x*	*y*	*z*	*U*_iso_*/*U*_eq_	
Sn1	0.5000	0.5000	0.5000	0.01202 (5)	
S1	0.36597 (3)	0.74692 (3)	0.61422 (5)	0.02012 (9)	
C1	0.46144 (12)	0.63979 (12)	0.6694 (2)	0.0140 (3)	
C2	0.50206 (12)	0.65832 (13)	0.8364 (2)	0.0134 (3)	
H2	0.5562	0.6088	0.8914	0.016*	
C3	0.45677 (14)	0.75770 (14)	0.9212 (2)	0.0154 (3)	
C4	0.38176 (14)	0.81447 (13)	0.8139 (2)	0.0192 (3)	
H4	0.3429	0.8832	0.8459	0.023*	
C5	0.48542 (16)	0.79485 (16)	1.1056 (2)	0.0206 (4)	
H5A	0.4919	0.8788	1.1097	0.031*	
H5B	0.5586	0.7602	1.1414	0.031*	
H5C	0.4244	0.7697	1.1859	0.031*	

### Atomic displacement parameters (Å^2^)

**Table:**

	*U*^11^	*U*^22^	*U*^33^	*U*^12^	*U*^13^	*U*^23^
Sn1	0.01360 (6)	0.01360 (6)	0.00885 (9)	0.000	0.000	0.000
S1	0.0221 (2)	0.0223 (2)	0.0160 (2)	0.00775 (16)	−0.00468 (16)	0.00016 (16)
C1	0.0138 (7)	0.0138 (7)	0.0144 (8)	0.0010 (5)	0.0011 (6)	0.0019 (6)
C2	0.0133 (6)	0.0140 (7)	0.0129 (8)	0.0000 (5)	0.0011 (6)	0.0020 (6)
C3	0.0165 (8)	0.0151 (8)	0.0146 (8)	−0.0017 (6)	0.0023 (6)	0.0011 (6)
C4	0.0220 (8)	0.0172 (8)	0.0185 (9)	0.0055 (6)	0.0014 (7)	−0.0008 (7)
C5	0.0267 (9)	0.0213 (9)	0.0139 (9)	−0.0005 (7)	0.0007 (7)	−0.0030 (7)

### Geometric parameters (Å, °)

**Table:**

Sn1—C1^i^	2.1209 (15)	C2—H2	0.95
Sn1—C1	2.1209 (15)	C3—C4	1.364 (2)
Sn1—C1^ii^	2.1209 (15)	C3—C5	1.502 (2)
Sn1—C1^iii^	2.1209 (15)	C4—H4	0.95
S1—C4	1.7173 (16)	C5—H5A	0.98
S1—C1	1.7206 (15)	C5—H5B	0.98
C1—C2	1.370 (2)	C5—H5C	0.98
C2—C3	1.424 (2)		
			
C1^i^—Sn1—C1	111.58 (4)	C4—C3—C2	111.07 (15)
C1^i^—Sn1—C1^ii^	105.32 (8)	C4—C3—C5	124.00 (16)
C1—Sn1—C1^ii^	111.58 (4)	C2—C3—C5	124.92 (16)
C1^i^—Sn1—C1^iii^	111.58 (4)	C3—C4—S1	111.98 (12)
C1—Sn1—C1^iii^	105.32 (8)	C3—C4—H4	124.0
C1^ii^—Sn1—C1^iii^	111.58 (4)	S1—C4—H4	124.0
C4—S1—C1	92.70 (8)	C3—C5—H5A	109.5
C2—C1—S1	109.51 (12)	C3—C5—H5B	109.5
C2—C1—Sn1	127.51 (11)	H5A—C5—H5B	109.5
S1—C1—Sn1	122.93 (8)	C3—C5—H5C	109.5
C1—C2—C3	114.74 (14)	H5A—C5—H5C	109.5
C1—C2—H2	122.6	H5B—C5—H5C	109.5
C3—C2—H2	122.6		
			
C4—S1—C1—C2	0.03 (12)	S1—C1—C2—C3	0.28 (17)
C4—S1—C1—Sn1	177.81 (9)	Sn1—C1—C2—C3	−177.37 (11)
C1^i^—Sn1—C1—C2	149.39 (12)	C1—C2—C3—C4	−0.54 (19)
C1^ii^—Sn1—C1—C2	−93.09 (10)	C1—C2—C3—C5	178.39 (14)
C1^iii^—Sn1—C1—C2	28.15 (11)	C2—C3—C4—S1	0.54 (17)
C1^i^—Sn1—C1—S1	−27.98 (10)	C5—C3—C4—S1	−178.40 (12)
C1^ii^—Sn1—C1—S1	89.54 (12)	C1—S1—C4—C3	−0.34 (13)
C1^iii^—Sn1—C1—S1	−149.22 (11)		

Symmetry codes: (i) −*y*+1, *x*, −*z*+1; (ii) *y*, −*x*+1, −*z*+1; (iii) −*x*+1, −*y*+1, *z*.
